# Nitrogen-corrected apparent metabolizable energy and standardized ileal amino acid digestibility of Brazilian DDGS for broilers

**DOI:** 10.1016/j.psj.2025.105271

**Published:** 2025-05-07

**Authors:** Bruno F. de Almeida, Ideraldo L. Lima, Kelly M.M. Dias, Carlos H. Oliveira, Kaique M. Gomes, Artur M. Ribeiro, Brian P. Mike, Arele A. Calderano

**Affiliations:** a Department of Animal Science, Federal University of Viçosa, Viçosa, Minas Gerais, Brazil; b IL Lima Consultoria em Nutrição Animal, Vila Velha, Espírito Santo, Brazil; c FS Bioenergia, Lucas do Rio Verde, Mato Grosso, Brazil

**Keywords:** Corn coproducts, Dried distiller’s grains with solubles, AMEn, SIAAD, Chicken

## Abstract

Metabolism and digestibility trials were performed on broiler chickens to determine the nitrogen-corrected apparent metabolizable energy (AMEn) values and the standardized ileal amino acids digestibility (SIAAD) coefficients for broiler chickens of dried distiller’s grains with solubles (DDGS) from three Brazilian processors. The metabolism and digestibility trials were performed on 20-30 day old chickens, with total excreta method used to determine energy values and the ileal digesta collection method used to determine SIAAD coefficients. The data obtained was subjected to analysis of variance and the means were compared using the Tukey test at 5 % significance. The AMEn values were 2,408.70 kcal/kg for DDGS 1, 1,763.20 kcal/kg for DDGS 2, and 1,873.40 kcal/kg for DDGS 3, on dry matter basis. The DDGS 1 had the highest AMEn value (P < 0.05). Regarding the SIAAD coefficients, DDGS 1 had the highest values for arginine, histidine, leucine, cystine, and proline (P < 0.05). In conclusion, there is variation in the nutritional value of DDGS available in Brazil.

## Introduction

Modern technological advances have enabled the incorporation of previously underutilized or discarded ingredients into animal feed, optimizing the recovery of nutrients and energy. As a result, co-products derived from industrial processes, such as biofuel production, have assumed a central role due to their nutritional value and potential to reduce animal production's environmental impact (Shurson, 2017).

Corn dried distiller’s grains with solubles (**DDGS**) are a co-product generated during corn ethanol production. It is utilized in broiler nutrition as an alternative feed ingredient which is a source of protein, essential amino acids (**AA**) and energy (Shurson, 2017; Dalólio et al., 2024). Additionally, DDGS are rich in dietary fiber and low in phytate content (Shurson, 2017; Dalólio et al., 2024). Compared to soy-bean meal (SBM), the main protein ingredient used in broiler nutrition, DDGS has a lower crude protein (CP) value. The CP of DDGS is approximately 30 % (Rochell et al., 2011; Dozier III et al., 2015), while in SBM it is approximately 45 % (Rostagno et al., 2017).

Ethanol production from corn involves many processors, and the resulting DDGS can exhibit considerable variation in chemical composition and amino acid digestibility due to differences in processing conditions, such as heat treatment (Rochell et al., 2011). In this context, nitrogen-corrected apparent metabolizable energy (**AMEn**) and the standardized ileal amino acids digestibility (**SIAAD**) of the DDGS sources are essential parameters for diet formulation..

The Midwest region of Brazil is the leading producer of corn ethanol in the country, home to 24 of the 29 operational corn ethanol plants. For this study, we selected three major processors in this region, collectively responsible for more than 50 % of Brazil’s corn ethanol production and significant contributors to the national supply of DDGS for animal feed. Therefore, this study aimed to determine the AMEn, and SIAAD values for broilers of corn DDGS from three Brazilian processors for use in broiler nutrition.

## Material and methods

### Ethics committee

All procedures adopted in this study were previously approved by the Ethics Committee in the Use of Farming Animals at the Federal University of Viçosa, under protocol number 119/2023, following the rules of the National Council for Experimentation Animal Control.

### Bird husbandry

Experiments were carried out at the Poultry Nutrition and Production Sector of the Department of Animal Science, Federal University of Viçosa, Viçosa, Minas Gerais, Brazil.

A total of 480 Cobb500™ male chicks were used in the experiments to determine AMEn and SIAAD. The birds were raised in protective circles equipped with tubular feeders and pendular drinkers with free access to fresh water and feed until they reached the ideal age for the trials. During this period, the birds received starter diets formulated according to the recommendations of Rostagno et al. (2017) and were reared according to Cobb500™ guideline recommendations.

### DDGS

The same batches of DDGS 1, DDGS 2, and DDGS 3 were used in the two experiments and were analyzed for their nutrient profile by CBO Análises Laboratoriais (Valinhos, São Paulo, Brazil). The analyzed compositions of each DDGS used in this study are shown in Table 1.

**Table 1:** Composition of the experimental diets for the metabolism and digestibility trials, and analyzed composition of DDGS sources.

Metabolizable energy trial		Amino acid digestibility trial		
Ingredients, %	Basal diet	Ingredients, %		NFD^3^
Corn	68.75	Starch		82.75
Soybean meal	25.24	Sugar		5.00
Soybean oil	2.07	Soybean oil		5.00
Dicalcium phosphate	1.54	Dicalcium phosphate		1.62
Limestone	0.75	Limestone		0.80
Salt	0.49	Salt		0.45
L-Lysine HCL, 78.8%	0.45	Corncob		3.00
DL-Methionine, 99%	0.27	Mineral Premix^2^		0.05
Vitaminic Premix^1^	0.13	Vitaminic Premix^1^		0.13
Mineral Premix^2^	0.12	Choline chloride, 60 %		0.20
Choline chloride, 60 %	0.10	Indigestible marker		1.00
L-Threonine, 98 %	0.09			
**Total**	**100.00**	**Total**		**100.00**

*Analyzed composition of DDGS sources*				
		DDGS 1	DDGS 2	DDGS 3
Dry matter, %		91.23	90.04	91.80
Crude protein, %		29.50	30.43	32.00
Gross energy, kcal/kg		4,544	4,717	4,554
Ether extract, %		8.88	7.77	8.45
Starch, %		4.05	3.20	2.80
Crude fiber, %		8.79	7.27	5.96
Neutral detergent fiber, %		45.93	43.88	39.70
Acid detergent fiber, %		17.80	15.14	15.87
Ash, %		3.09	3.71	4.24
Acid detergent insoluble nitrogen, %		0.40	0.40	0.56
Calcium, %		0.01	0.01	0.06
Total phosphorus, %		0.53	0.61	0.68
Sodium, %		0.09	0.06	0.06
Selenium, mg/kg		0.05	0.02	0.02

1 Vitamin premix provided per kg of product: vitamin A, 9,375,000 IU; vitamin D3, 2,375,000 IU; vitamin E, 35,000 IU; vitamin B1, 2.50 mg; vitamin B2, 6.25 mg; vitamin B6, 3.5 mg; vitamin B12, 0.015 mg; vitamin K3, 1.88 mg; pantothenic acid, 12.5 mg; nicotinic acid, 37.50 mg; folic acid, 0.875 mg; biotin, 0.088 mg.

2 Trace mineral premix provided per kg of product: Mn, 88 mg; Fe, 62.5 mg; Zn, 81,3 mg; Cu, 12.5 mg; I, 1.25 mg; Se, 0.375 mg.

3 NFD = N-free diet

### Metabolism trial – AMEn

A total of 240 birds, each 20 days old, were randomly assigned to metabolic cages (600 cm²/bird) into 4 treatments with 10 replicates and 6 birds per experimental unit. Each cage was equipped with a nipple drinker and a trough feeder to guarantee free access to water and feed during the experimental period.

The treatments consisted of a basal diet (**BD**, Table 1) and three diets composed of 70 % BD and 30 % of each DDGS source.

The experimental period was 10 days. The first 5 days were used to adapt the birds to the experimental diets, and the final 5 days were for the excreta collection period. Excreta were collected twice a day, at 8 a.m. and 4 p.m., stored in identified plastic bags for each experimental unit, and kept in a freezer at -20 °C during the collection period until further analysis. At the end of the last collection day, excreta were defrosted, weighed, and homogenized to take a representative sample from each experimental unit. They were arranged into 200 g samples, lyophilized at -40 °C for 72 hours, and ground in a ball mill (Tecnal Equipamentos para Laboratório. TE-350, São Paulo, Brazil) for five minutes until a fine and homogeneous mixture was formed.

Homogenous and representative excreta samples, experimental diets, and DDGS sources were analyzed to dry matter (**DM**), nitrogen, and gross energy (**GE**) by CBO Análises Laboratoriais (Valinhos, São Paulo, Brazil). The nitrogen balance (**NB**) was calculated by subtracting the amount of nitrogen excreted from the amount of nitrogen intake. The AME and AMEn values of each experimental diet were calculated using the following equation:

AME=(GEintake−GEexcretion)/DMintake

AMEn=[(GEintake−GEexcretion)−(8.22×NB)]/DMintake

The equation below was used to calculate the AMEn of DDGS sources:

AMEn=BD−[(BD−TD)/g/gofsubstitution]

BD is the AMEn of the basal diet and TD is the AMEn of each experimental diet with DDGS sources.

### Digestibility trial - SIAAD

A total of 240 birds, each 25 days old, were randomly assigned to metallic cages (600 cm²/bird) into 4 treatments, with 10 replicates and 6 birds per experimental unit. Each cage was equipped with a nipple drinker and a trough feeder to guarantee free access to water and feed during the experimental period.

The treatments consisted of a N-free diet (NFD) used to measure basal ileal endogenous AA losses and three diets with 30 % of either DDGS source replacing starch in the NFD. In all diets, 1 % Celite™ (acid-insoluble ash – **AIA**) was added as an indigestible marker (Table 1).

The birds were fed the experimental diets for 5 days and then slaughtered by cervical dislocation to collect the ileal digesta. The ileum was exposed through the abdominal incision and all its contents were collected and stored in plastic pots identified according to the respective experimental units. Before laboratory analysis, the collected digesta was lyophilized at -40 °C for 72 hours and ground in a ball mill. Samples of digesta and diets were analyzed for DM and ash content by CBO Análises Laboratoriais (Valinhos, São Paulo, Brazil). Moreover, DDGS sources, diets, and digesta were analyzed for AA and nitrogen content by CBO Análises Laboratoriais (Valinhos, São Paulo, Brazil).

Apparent and standardized ileal amino acids digestibility values and endogenous ileal losses were calculated using the equations below:

AIAADvalue(%)=[(AAdiet(DM,%)/AIAdiet(%))−(AAdigesta(%)−AIAdigesta(%))×100]/(AAdiet(DM,%)/AIAdiet(%))

IEAA(g/kgDM)=(AAdigesta(g/kgDM))x(AIAdiet(g/kgDM))/(AIAdigesta(g/kgDM))

SIAADvalue(%)=AIAADvalue(%)+(IEAA(g/kgDM)/AAdiet(g/kgDM)x100

AIAAD is the apparent ileal amino acid digestibility value, and IEAA is the ileal endogenous amino acids losses. The SIAAD concentration was calculated by multiplying each SIAAD value by the respective AA content in each ingredient.

### Statistical analysis

The data were analyzed using the GLM procedure in SAS version 9.4. The Bartlett and Shapiro-Wilk tests were used to check the homogeneity of variance and normality, respectively. An analysis of variance was then performed to test for differences among the treatments. The Tukey test was conducted at a 5 % significance level to determine significant differences between the treatments.

## Results and discussion

The determined values of AME and AMEn for DDGS sources are presented in Table 2. The AMEn values for DDGS 1, DDGS 2, and DDGS 3 are, respectively, 2,409 kcal/kg, 1,763 kcal/kg, and 1,873 kcal/kg on a DM basis and 2,198 kcal/kg, 1,558 kcal/kg and 1,720 kcal/kg on an as-fed basis. The higher energy values observed for DDGS 1 can be attributed to its greater fat and residual starch contents, as these nutrients are directly involved in energy provision. The AMEn values observed for the three DDGS sources were lower than those reported by Dalólio et al. (2024) and Rochell et al. (2011), who observed AMEn of 3,398 kcal/kg and 3,098 kcal/kg on a DM basis, respectively. These differences can be explained by the higher fat and starch contents in the DDGS referenced, compared with those evaluated in the present study. Additionally, the referenced DDGS contained lower neutral detergent fiber (NDF) levels than those reported here. These authors associated fat, starch, and fiber contents with energy values, supporting the interpretation of our findings. In contrast, the AMEn values reported here were similar to those reported by Rochell et al. (2011), Santos et al. (2019) and Moreno et al. (2023), who found values of 2,146 kcal/kg, 2,614 kcal/kg, and 2,476 kcal/kg, respectively. In these cases, the referenced DDGS had lower fat and residual starch content compared to our tested products, which may explain the reduced AMEn values. Shurson (2017) stated that some DDGS exhibit reduced fat content due to processing steps, particularly oil extraction, which leads to lower ether extract (EE) values and increased variability in energy content. These variations reinforce the idea that processing conditions, such as oil extraction methods or drying temperatures, can significantly influence the final energy content of DDGS. Moreover, the elevated ADF and NDF contents observed in our samples suggest reduced nutrient digestibility, especially in combination with lower fat and residual starch contents. Therefore, the variability in AMEn values among the DDGS sources evaluated can be attributed to differences in both chemical composition and industrial processing conditions, which are often not standardized across facilities.

**Table 2:** Apparent metabolizable energy (AME), nitrogen-corrected apparent metabolizable energy (AMEn), standardized ileal amino acid digestibility value (SIAAD), total amino acids content (AA), and standardized ileal digestibility (SID) on an as-fed basis of DDGS sources used in the metabolism and digestibility trials.

	DDGS 1			DDGS 2			DDGS 3			SEM¹	P-value
*Dry matter*											
AME, kcal/kg	2,608 a			1,929 b			2,014 b			91.92	0.002
AMEn, kcal/kg	2,409 a			1,763 b			1,737 b			84.65	0.002
*As-fed basis*											
AME, kcal/kg	2,380 a			1,737 b			1,848 b			84.43	0.002
AMEn, kcal/kg	2,198 a			1,588 b			1,720 b			77.82	0.001

	SIAAD, %			AA, %			SID			SEM¹	P-value
	DDGS 1	DDGS 2	DDGS 3	DDGS 1	DDGS 2	DDGS 3	DDGS 1	DDGS 2	DDGS 3		
*Essential AAs*											
Lysine	71.3	71.2	64.9	0.98	0.90	1.09	0.70	0.64	0.71	1.34	0.076
Methionine	84.6 a	84.4 a	79.3 b	0.44	0.48	0.45	0.37	0.40	0.36	0.83	0.003
Threonine	71.0 a	69.3 ab	63.7 b	1.12	1.14	1.28	0.80	0.79	0.82	1.23	<0.001
Arginine	83.0 a	80.9 b	77.2 b	1.37	1.34	1.54	1.14	1.08	1.19	0.85	0.002
Histidine	83.3 a	79.7 b	82.3 b	0.86	0.86	0.97	0.72	0.69	0.70	1.35	<0.001
Isoleucine	77.6 a	76.9 a	69.4 b	1.10	1.12	1.27	0.85	0.86	0.88	1.29	0.002
Leucine	87.5 a	85.9 b	79.9 c	3.34	3.47	3.72	2.92	2.98	2.97	1.08	<0.001
Valine	79.1 a	78.3 a	68.0 b	1.47	1.50	1.67	1.16	1.17	1.14	1.54	<0.001
Phenylalanine	84.6 a	84.1 a	76.7 b	1.35	1.40	1.54	1.14	1.18	1.18	1.11	<0.001
*Nonessential AAs*											
Alanine	85.2 a	84.1 a	77.4 b	2.08	2.18	2.39	1.77	1.83	1.85	1.04	<0.001
Aspartic acid	74.3 a	72.5 a	64.0 b	1.93	2.02	2.31	1.43	1.47	1.48	1.44	<0.001
Cysteine	80.8 a	70.0 b	66.5 b	0.50	0.43	0.46	0.40	0.30	0.31	1.78	<0.001
Glutamic acid	87.0 a	85.3 a	78.1 b	4.90	5.27	5.75	4.26	4.50	4.49	1.13	<0.001
Glycine	70.6 a	69.7 a	59.8 b	1.15	1.16	1.62	0.81	0.81	0.97	1.49	<0.001
Serine	79.3 a	77.1 a	69.8 b	1.45	1.51	1.63	1.15	1.16	1.14	1.25	<0.001
Tyrosine	83.2 a	82.7 a	74.6 b	1.12	1.15	1.15	0.93	0.95	0.95	1.21	<0.001
Proline	84.6 a	79.3 b	72.9 c	2.50	2.48	3.05	2.11	1.97	2.22	1.55	<0.001

Means in the same line with different letters differ significantly (P < 0.05).

¹SEM = standard error of mean

SID = (total amino acids concentration x standardized digestibility coefficient) / 100.

The results for SIAAD are also presented in Table 2. DDGS 3 exhibited the lowest SIAAD values for all AA analyzed, except for Lys, compared with DDGS 1 (P < 0.05). Furthermore, DDGS 1 had the highest SIAAD values for arginine, histidine, leucine, cysteine, and proline compared with DDGS 2 (P < 0.05). Heat damage may reduce amino acid digestibility in DDGS by increasing the risk of protein damage during ethanol production and the drying process (Almeida et al. 2013; Böttger and Südekum, 2018). Besides drying, further processing steps that involve high temperatures may affect the protein quality of DDGS (Böttger and Südekum, 2018). The variation in AA digestibility is often attributed to Maillard reactions between AA, especially lysine, and reducing sugars during the DDGS drying process (Almeida et al. 2013; Böttger and Südekum, 2018). We believe that heat damage is the primary factor influencing amino acid digestibility in the DDGS samples studied, as it is directly linked to Maillard reactions, which occur during ethanol production and drying. Variability may also be partly explained by differences in acid detergent insoluble nitrogen (**ADIN**) content. High ADIN concentrations are associated with reduced protein quality and amino acid digestibility (Almeida et al., 2013; Böttger and Südekum, 2018). During the DDGS process, heating may affect plant cell walls, including the binding of N compounds to fiber and the formation of artifact lignin polymers, which leads to high contents ADIN. Increased binding of N to plant cell walls could reduce precaecal (often termed ileal) N digestibility due to a lack of fibrolytic enzymes (Böttger and Südekum, 2018). Additionally, Dozier III et al. (2015) reported that fat content is positively correlated with amino acid digestibility. According to these authors, oil extraction during DDGS processing can expose amino acids to mechanical stress, thereby reducing digestibility. The total amino acid contents found in this study are consistent with previous reports. Compared with the values reported by NRC (1994), the DDGS evaluated here had higher concentrations of essential amino acids such as Lys, Thr, Val, and Arg (0.75 %, 0.92 %, 1.30 %, and 0.89 % on an as-fed basis, respectively). In contrast, the total Met content reported by the NRC (1994) was 0.60 %, which is higher than that found in our study. However, Dalólio et al (2024) reported a methionine concentration of 0.44 % (as-fed basis), consistent with the values observed in our study.

In conclusion, our results emphasize the variability in energy values and amino acid digestibility for broilers of Brazilian DDGS processors, mainly due to differences in composition and processing. Routine analysis of nutritional composition is essential to ensure accurate diet formulation. Given the high nutritional demands during the starter phase, DDGS should be used with caution in starter diets, preferring its incorporation during the grower and finisher phases, unless the quality of the batch is assured.

## Declaration of competing interest

The authors declare that they have no known competing financial interests or personal relationships that could have appeared to influence the work reported in the paper “Research Note: Nitrogen-corrected apparent metabolizable energy and standardized ileal amino acid digestibility of Brazilian DDGS for broilers”.
